# Cross-Reactivity of Herpesvirus-Specific CD8 T Cell Lines Toward Allogeneic Class I MHC Molecules

**DOI:** 10.1371/journal.pone.0012120

**Published:** 2010-08-12

**Authors:** Alexis Morice, Béatrice Charreau, Bérangère Neveu, Sophie Brouard, Jean-Paul Soulillou, Marc Bonneville, Elisabeth Houssaint, Nicolas Degauque

**Affiliations:** 1 UMR892, INSERM - Institut de Recherche Thérapeutique de l'Université de Nantes, Nantes, France; 2 UMR 643, INSERM, Nantes, France; 3 ITUN, CHU Nantes, Nantes, France; 4 Faculté de Médecine, Université de Nantes, Nantes, France; 5 Faculté des Sciences, Université de Nantes, Nantes, France; Singapore Immunology Network, Singapore

## Abstract

Although association between persistent viral infection and allograft rejection is well characterized, few examples of T-cell cross-reactivity between self-MHC/viral and allogeneic HLA molecules have been documented so far. We appraised in this study the alloreactivity of CD8 T cell lines specific for immunodominant epitopes from human cytomegalovirus (HCMV) and Epstein-Barr virus (EBV). CD8 T cell lines were generated after sorting with immunomagnetic beads coated with either pp65_495–503_/A*0201, BMLF1_259–267_/A*0201, or BZLF1_54–64_/B*3501 multimeric complexes. Alloreactivity of the CD8 T cell lines against allogeneic class I MHC alleles was assessed by screening of (i) TNF-α production against COS-7 cells transfected with as many as 39 individual HLA class I-encoding cDNA, and (ii) cytotoxicity activity toward a large panel of HLA-typed EBV-transformed B lymphoblastoid cell lines. We identified several cross-reactive pp65/A*0201-specific T cell lines toward allogeneic HLA-A*3001, A*3101, or A*3201. Moreover, we described here cross-recognition of HLA-Cw*0602 by BZLF1/B*3501-specific T cells. It is noteworthy that these alloreactive CD8 T cell lines showed efficient recognition of endothelial cells expressing the relevant HLA class I allele, with high level TNF-α production and cytotoxicity activity. Taken together, our data support the notion that herpes virus-specific T cells recognizing allo-HLA alleles may promote solid organ rejection.

## Introduction

It is now well established that the memory subset of circulating T cells contribute to alloresponse, thus explaining that viral infections are associated with graft failure in human transplant recipients [1], [2], [3]. A range of acute viral infections, most particularly cytomegalovirus (HCMV) infection, has been linked with initiating the clinical complications that often follow transplantation [4]. The best evidence that HCMV is involved in acute and chronic rejection is based on studies with the anti-HCMV drug ganciclovir in humans and animal models that demonstrate a reduction in allograft failure in solid organ transplant patients [5]. HCMV could account for graft rejection by selective endothelial cell activation thereby attracting and activating alloreactive T cells [2]. Another factor of the association between HCMV infection and allograft graft rejection could be cross-reactivity of HCMV-specific T cells to allogeneic HLA molecules. Persistent viral infections have a profound impact on T cell repertoire, since they lead to long-term clonal expansions of virus-specific memory CD8 T cells. Large clonal expansions of αβ T cells within the human peripheral repertoire have been documented in several acute viral infections [6] and in healthy individuals [7]. In particular, human CD8 memory T cell repertoire is often dramatically skewed by predominant clones directed against HCMV or Epstein-Barr virus (EBV), which can persist unaltered for many years [8], [9], [10]. Through cross-reactivity, these memory T cells could contribute to the alloresponse, owing to their lack of requirement for co-stimulation, easy and rapid activation, and vigorous effector functions [11].

Though association between persistent viral infection and allograft rejection is well admitted, few examples of T-cell cross-reactivity between self-MHC/viral and allogeneic HLA molecules have been documented so far. The influence of antiviral T cell responses on the CD8^+^ T cell alloreactive repertoire was first described for an EBV T cell response specific to the EBNA3A_325–333_/B*0801 EBV epitope [12], [13], [14]. More recently, cross-reactivity of HCMV-specific and herpes simplex virus-specific CD8 T cells to allogeneic HLA alleles has been reported [15], [16].

To appraise the contribution of EBV- or HCMV-specific CD8 T cell responses to the allogeneic repertoire, we screened a number of CD8 T cell lines, that had been sorted with recombinant peptide/MHC class I (pMHC) multimeric complexes, on a large panel of HLA class I alleles expressed either by transfected COS cells or by EBV-transformed B lymphoblastoid cell lines (LCL) for cross-reactivity to allogeneic class I HLA molecules. Our study was focused on the pp65_495–503_/A*0201 HCMV epitope (NLVPMVATV) [17] and two epitopes of early lytic EBV proteins (BZLF1_54–64_/B*3501: EPLPQGQLTAY [18], [19] and BMLF1_259–267_/A*0201: GLCTLVAML [20], [21]), for which immunodominance [17], [19], [22], [23] and high frequency [10], [24] is well documented. This unveiled several allospecific CD8 T cell responses, leading to cytotoxicity and TNF-α production against primary endothelial cell cultures expressing the relevant allogeneic HLA alleles. This might have important physiopathological implications in an allograft setting, which are discussed.

## Results

### Screening of HCMV- or EBV-specific CD8 T cell lines for cross-recognition of allogeneic MHC molecules

**Figure 1:**
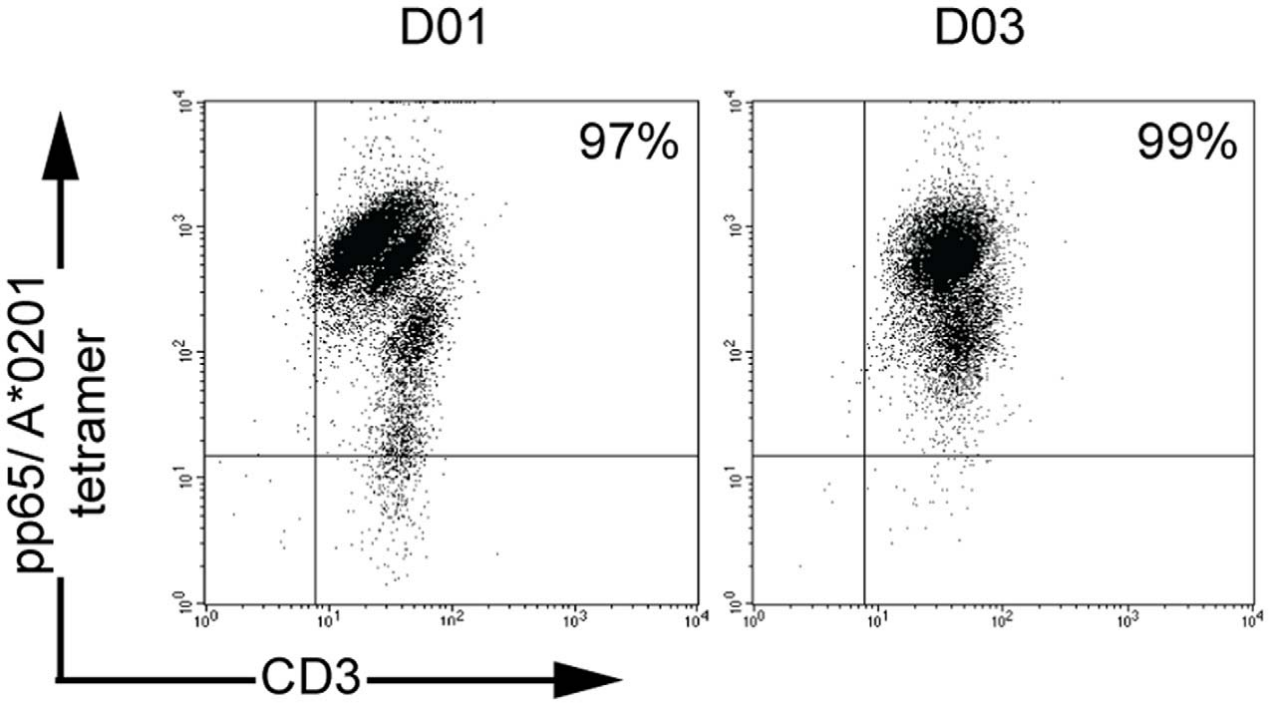
Enrichment in pp65_495–503_/A*0201- or BZLF1_54–64_/B*3501-specific T cells after sorting of CD8 T cells with pMHC magnetic multimers. (**A**) CD8 T cells, derived from the PBL from D01 and D03 were sorted with 245V mutated pp65_495–503_/A*0201 multimers, then expanded in culture, and stained with PE-conjugated pp65_495–503_/A*0201 tetramers and FITC-conjugated anti-CD3. (**B**) CD8 T cells derived from the PBL from D15 were sorted with 245V mutated BZLF1_54–64_/B*3501 multimers, and then expanded in culture. Unsorted and sorted T cells were stained with PE-conjugated BZLF1/B35 tetramers and FITC-conjugated anti-CD3. The percentage of positive cells is indicated in the upper right quadrant.

To assess the influence of CD8 T cell responses specific to HCMV or EBV to the allogeneic repertoire, we screened CD8 T cell lines sorted with recombinant pMHC multimeric complexes specific to HCMV (pp65_495–503_/A*0201) or EBV (BMLF1_259–267_/A*0201 or BZLF1_54–64_/B*3501) epitopes for cross-recognition of allogeneic MHC class I molecules, taking into account the immunodominance of those responses and the frequent expression of A*0201 and B*3501 alleles (Table 1). Most T cell lines analyzed were derived from PBL from healthy donors (D01 to D08, D12). Other T cell lines were derived from PBL from patients suffering from arthritis (D09 to D11, D13 to D16). The enrichment in EBV- or HCMV-specific T cells was checked by staining by ad hoc pMHC tetramers, and two successive sortings were made, when necessary, to achieve a purity between 89 to 100% (Table 1, Fig. 1.A, and data not shown). Staining of T cell lines before sorting indicated frequencies comprised between 0.1 and 5.6% (Fig. 1.B and data not shown). Two unsorted T cell lines, derived from A2-negative donors were also included in the screening.

**Table 1:** Screening of CD8 T cell lines enriched in HCMV- or EBV-specific T cells for cross-reactivity to allogeneic MHC molecules.

Donor#	Molecular HLA class I typing						% of tetramer-positive T cells	Reactivity to allogeneic HLA^a^
	HLA-A*		HLA-B*		HLA-Cw*			
pp65/A*0201-sorted T cell lines								
D01	0201	0301	3501	4402	0401	0501	97	A*3101
D02	0201	33	08	14	07	08	98	none
D03	0201	0201	1302	5101	0202	0602	99	A*3201
D04	0201	ND	ND		ND		98	none
D05	0201	ND	ND		ND		99	none
D06	0201	ND	ND		ND		99	none
D07	0201	2301	2705	4402	0102	0509	98	none
D08	0201	2301	4101	4402	0501	1701	89	A*3001
D09	0201	30	08	44	04	07	98	none
D10	0201	0201	1501	4403	0304	0501	95	none
D11	0201	1101	0801	2705	01	07	97	none
BMLF1/A*0201-sorted T cell lines								
D12	0201	3201	15	15	0303	04	93	none
D13	0201	0201	2705	4002	0102	1501	95	none
BZLF1/B*3501-sorted T cell lines								
D14	0301	1101	0701	3501	04	07	92	none
D15	2402	3101	3501	4001	03	07	97	Cw*0602
D16	2402	3201	2705	3501	01	04	97	none

a CD8 T cell lines were screened on COS-7 cells transfected with individual HLA-encoding cDNA and TNF-α production was measured after a 6h-coculture. All T cell lines were PBL-derived. ND : not determined.

T cell lines were screened both for TNF-α production against COS-7 cells transfected with as many as 39 individual HLA class I-encoding cDNA, and for cytotoxicity toward a panel of 30 HLA-typed LCLs (Table 2), in order to detect allo-MHC recognition. Data are summarized in Table 1.

**Table 2:** HLA class I typing of LCL.

LCL	HLA-A*		HLA-B*		HLA-Cw*	
ADA	0201		2705	4002	0102	1502
AK1	0201	0301	0702	1402	0702	0802
**BAR**	**3001**	2402	4403	5101	0602	1601
BAX	0101	0301	0702	1501	0303	0702
**BOI**	2402	**3101**	3501	4001	03	07
COL	0102		0801	3501	0401	07
CRE	0101	2301	0801	4403	0701	0401
D1	0201	0301	3501	4402	0401	0501
D2	0201	33	08	14	07	08
D45	0201	2902	0702	0801	0701	0702
DAB	0201	1101	1801	5501	03	07
DUC	3002		1801		0501	
GAS	0301	2402	2705	5101	0202	1602
HOB	0101		0801		0701	
HVI	0101	0201	0801	1501	0304	0701
JES	0201		2705		01	
**JHAF**	**3101**		5101		08	
KER	0101	6801	0801	5101	07	15
LEE	0301		15	27	ND	
LEP	0201	2501	1801	4402	0501	1203
MHE	0201	33	14	57	0602	0801
NIJ	0201	33	08	58	0302	0304
PAU	0201		1801	4001	0304	1203
PIP	0102	0201	5701	4002	ND	
**SBN**	0201	**3201**	1402	5701	0602	0802
SYL	0301		0801	1801	0701	
TAY	0101	1101	0702	3501	0401	0702
TIE	0201	2402	2705	35	0202	0401
TIM	0201	2601	2703	4501	0102	0602
YOU	0102	2402	2702	3501	ND	

ND : Not determined. LCL and the HLA triggering allorecognition are indicated in bold.

### pp65_495–503_/A*0201 epitope specific T cells exhibit alloreactivity against HLA-A*3001, A*3101, or A*3201

**Figure 2:**
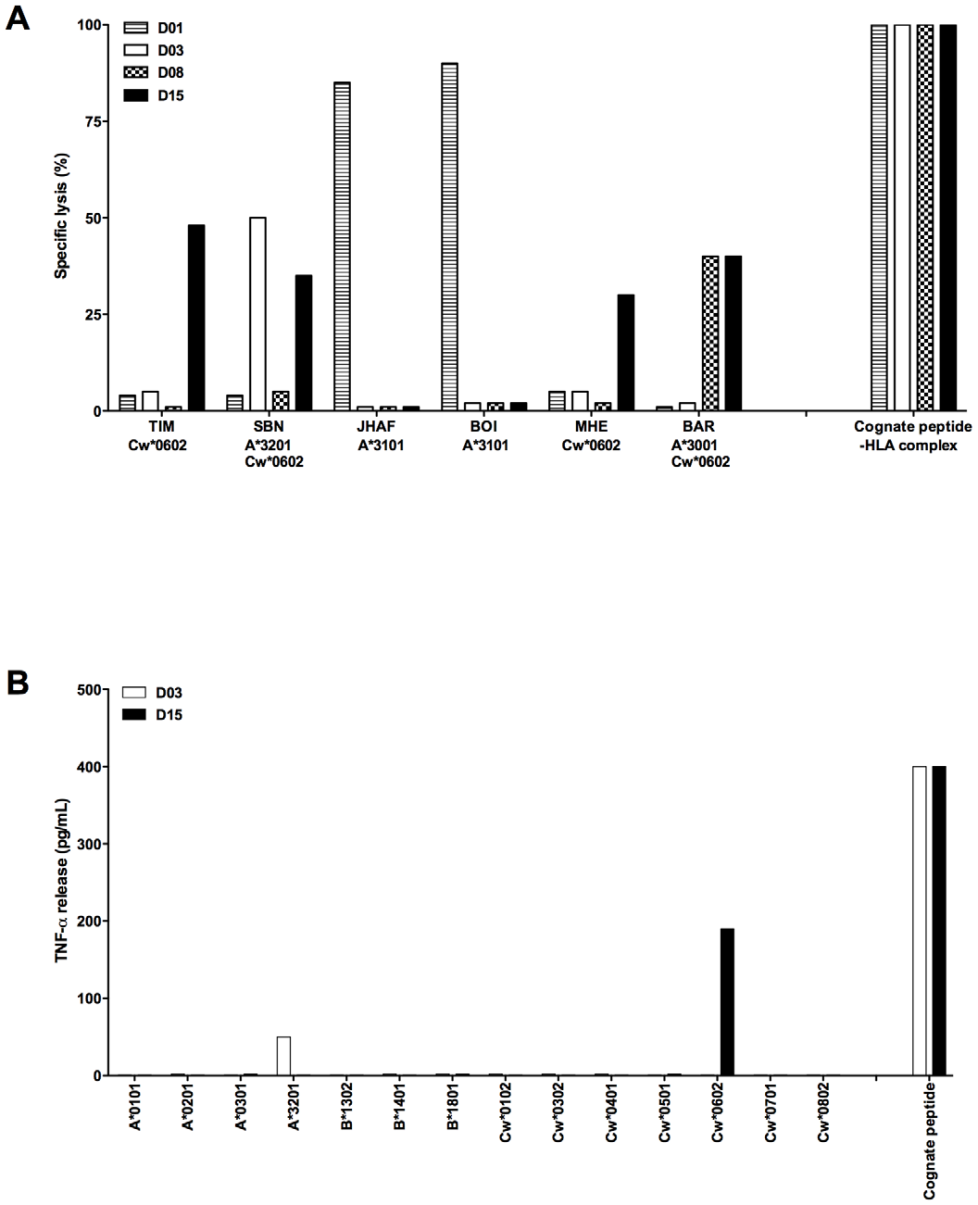
Screening of HCMV- or EBV-specific CD8 T cell lines for cross-recognition of allogeneic MHC molecules. CD8 T cell lines, sorted with recombinant pMHC multimeric complexes specific to HCMV (pp65_495–503_/A*0201) or EBV lytic epitopes (BMLF1_259–267_/A*0201 or BZLF1_54–64_/B*3501), were tested: (A) for cytotoxicity toward a panel of 30 HLA-typed LCL, in a 4-h chromium release assay (Effector-Target ratio 15∶1). HLA-specific killing of LCL was observed for 3 of the 11 pp65/A2-specific T cell lines tested: T cell line from D01 killed A*3101^+^ LCL, T cell line from D03 killed A*3201^+^ LCL, and T cell line from D08 killed A*3001^+^ LCL. The BZLF1/B*3501-sorted T cell line from D15 killed LCL sharing Cw*0602 expression. Only T cell lines that showed alloresponse are displayed. LCL triggering an alloresponse are shown as well as some of the tested LCL which do not elicit a cytotoxic response. Cognate peptide-HLA complexes (pp65_495–503_/A*0201 or BZLF1_54–64_/B*3501) were used as positive control. Data are presented as the mean percentage lysis and are representative of 3 different experiments. (**B**) for TNF-α production toward COS-7 cells transfected with plasmids encoding class I HLA alleles. T cells were added 2 days after the transfection, and the TNF-α content of the supernatant, expressed in pg/mL, was estimated 6 h later by testing the toxicity of the supernatants for TNF-α sensitive WEHI-164 clone 13 cells. TNF-α production was observed for the pp65/A*0201-sorted T cell line from D03, that recognized selectively COS cells expressing HLA-A*3201, and for the BZLF1/B*3501 T cell line from D15 that recognized selectively COS cells expressing HLA-Cw*0602. Plasmids encoding class I HLA alleles that elicits a response are shown as well as some of the plasmid encoding class I HLA alleles that do not induce TNF-α secretion. T cell lines that did not produce TNF-α are not represented. Cognate peptide-HLA complexes (pp65_495–503_/A*0201 or BZLF1_54–64_/B*3501) were used as positive control. One out three independent experiments is shown.

Cross-reactivity to allogeneic HLA molecules was found for 3 out of 11 pp65/A2-sorted T cell lines, each one recognizing a different HLA-A allele. The T cell line derived from D03 killed only the LCL SBN, the only one of the panel to express A*3201. D01-derived T cell line killed LCL BOI and JHAF, sharing A*3101 expression, and D08-derived T cell line killed LCL BAR, the only one to express A*3001, but did not kill LCL DUC A*3002^+^ (Fig. 2A). The percentage of killing varied from 40 to 90%, probably reflecting the representation of the alloreactive T cells within the polyclonal T cell line. In the COS transfection assay, TNF-α production was observed only for the T cell line derived from D03, that responded to COS cells expressing A*3201 (Fig. 2B). The cDNAs encoding HLA-A*3101 and -A*3001 were not available, thus explaining that no response was observed for the T cell lines derived from D01 and D08 in the COS assay. Positive controls indicated that all pp65/A2-sorted T cell lines killed LCL A*0201^+^ loaded with the pp65_495–503_ peptide at 1 µM, with 80% to 90% killing efficiency and produced high level TNF-α against COS cells co-expressing HLA-A*0201 and pp65 (data not shown). Two unsorted T cell lines, derived from HLA-A*0201-negative donors were included in the screening. They showed allorecognition of HLA-A*0201, producing TNF-a selectively against COS cells expressing A*0201, and killing all A*0201^+^ LCL (data not shown). This indicates that results obtained from the two assays were fully consistent, thus validating the use of COS cells transfected with individual HLA allele to screen for allorecognition in the MHC class I context.

To appraise the polyclonality of the pp65/A2-specific T cell lines, their TCR Vβ repertoire was analyzed by immunofluorescence, using Vβ-specific mAb (Table 3). T cell lines derived from healthy donors used an heterogeneous set of TCR Vβ families. In contrast, T cell lines derived from RA patient PBL, that were analyzed in a previous study [25], showed a dramatic skewing of their TCR Vβ repertoire, as >95% of T cells for a given donor expressed the same Vβ region. Three TCR Vβ regions (TRBV10-3, TRBV12-4 and TRBV20-1) were preferentially expressed by most of the pp65/A2-specific T cell lines analyzed in the present study.

**Table 3:** Analysis of the TCR Vb repertoire of pp65_495–502_/A*0201- or BZLF1/B*3501-sorted T cell lines, using TCR Vβ-specific mAb.

TCR Vβ specific mAb																								
TRBV	2 (22)	3-1 (9)	4-1 (7.1)	4-3 (7.2)	5-1 (5.1)	5-5 (5.3)	5-6 (5.2)	6-2 (13.2)	6-6 (13.6)	6-9 (13.1)	9 (1)	10-3 (12)	11-2 (21.3)	12-4 (8)	13 (23)	14 (16)	18 (18)	19 (17)	20-1 (2)	25-1 (11)	27 (14)	28 (3)	29-1 (4)	30 (20)
pp65/A*0201-sorted T cell lines																								
** D01**								**18.2**		**57.4**									**10.6**		**12.3**			
D02			1.6											8.2							89.2			
** D03**				**1**	**1.1**			**2**	**2.2**	**20.8**	**2.2**	**1.3**	**6.9**	**10.3**				**1.1**		**1.2**	**13.2**	**2.7**		
D04										91.5	6.1													
D05	6.9					1.1		2		20.9	5.3			2.4	1				6.8	24.7	2.3			
D06						9.5	3.4	2.3			41.4	1.3	1.4	11.7				27.7						
D07				28.4						21.1	3.9			8.7			12.1	1.6	2.5		10.2			
** D08**					**1.8**					**1.7**	**2.6**			**42.3**				**1**	**8.5**					
D09																					100^a^			
D10																					99.0			
D11										100^a^														
BZLF1/B*3501-sorted T cell lines																								
D14					1.1			2.9				48.8		2.3					36.6			5.2		
** D15**			**1.2**									**96.4**												
D16			3.9									55.2		26.3								16.4		

The percentage of pp65/A*0201- or BZLF1/B*3501-specific T cells stained by the various anti-TCR Vβ mAb is mentioned according to the IMGT nomenclature (Beckman Coulter anti-TCR Vβ name is indicated in bracket). All T cell lines were derived from PBL, either from healthy donors or from RA patients.

a Percentage determined by TCR sequencing (ref. 28). T cell lines exhibiting alloreactivity are marked in bold.

**Figure 3:**
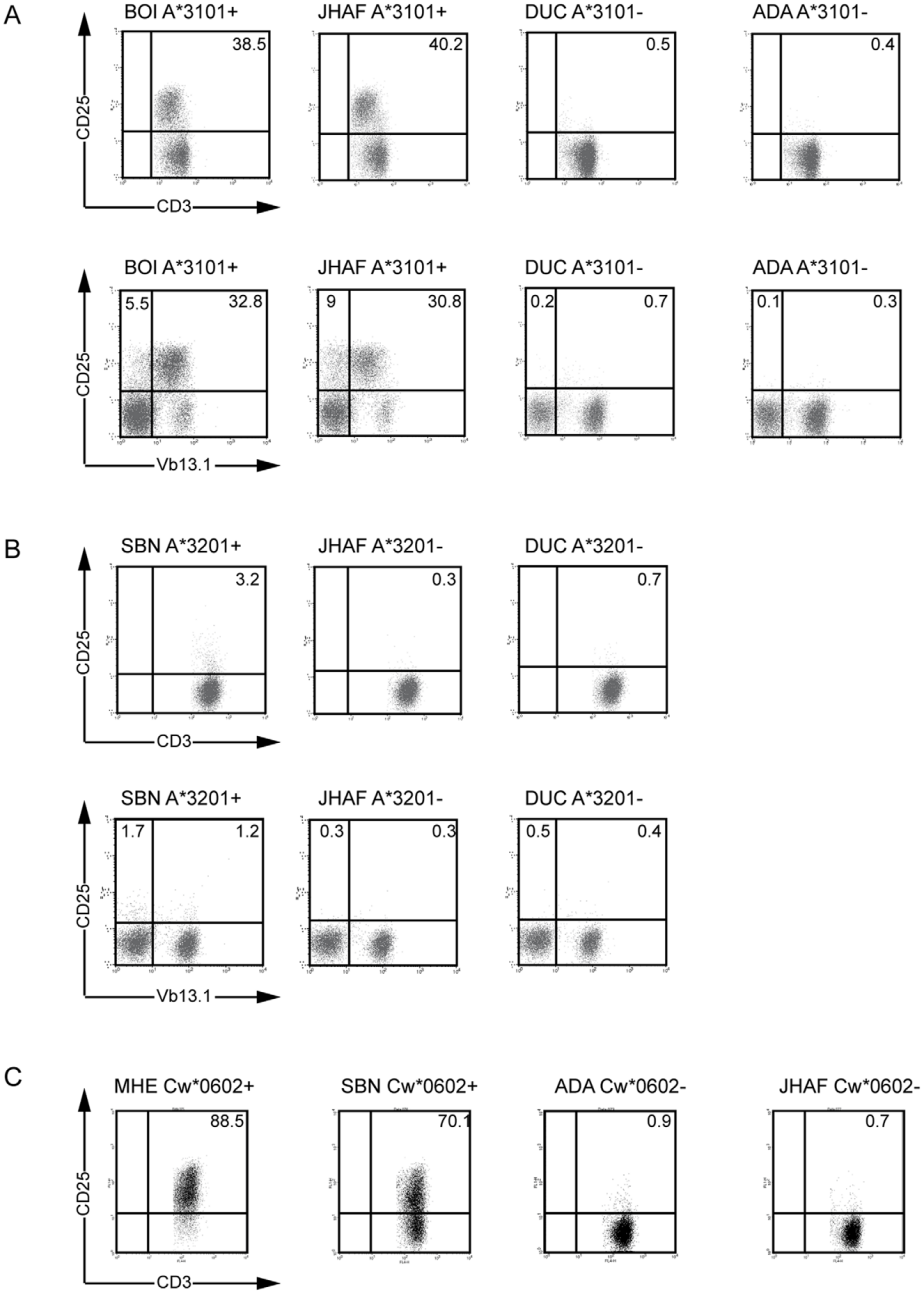
Estimation of the percentage of alloreactive T cells within T cell lines sorted with pMHC multimers. T cell lines from D01, alloreactive to A*3101 (**A**), from D03, alloreactive to A*3201 (**B**), and from D15, alloreactive to Cw*0602 (**C**) were stimulated with allogeneic LCL cells expressing, or not, the relevant allogeneic HLA allele (E∶T = 1∶1). Sixteen hours later, cells were double stained by anti-CD25-FITC and anti-CD3-APC mAb. LCL expressing the relevant allogeneic HLA molecules induced CD25 expression by alloreactive T cells. The % of CD3^+^ T cells stained for CD25 is indicated. T cell lines from D01 and D03 were triple stained by anti-CD3-APC (for gating T cells), anti-CD25-FITC and anti-Vβ-PE mAb and analysed by flow cytometry using a BD FACSCalibur (Becton Dickinson, san Jose, CA). Data are shown for anti-Vβ13.1 (TRBV6-9), only, as cells double stained by anti-CD25 and other anti-Vβ were not found. The % of CD3^+^ T cells stained for CD25 or for the TCR Vβ13.1 (TRBV6-9) region is indicated. Data are representative of 3 different experiments.

To assess the percentage of alloreactive T cells within a given pp65/A2-sorted T cell line and the TCR Vβ region used by the fraction of alloreactive T cells, we conducted a CD25 upregulation assay. T cell lines were stimulated overnight with LCL expressing, or not, the relevant allogeneic HLA allele and cells were triple stained by anti-CD3-APC, anti-CD25-FITC and by anti-Vβ-PE mAb specific to each one of the TCR Vβ used by this T cell line, as indicated in Table 3. Such an analysis was done for the T cell lines from D01 and D03. Data indicated that 40% of T cells within the pp65/A2-specific T cell line from D01 responded to LCL expressing A*3101 (Fig. 3A). Intracellular IFN-γ detection was also performed after stimulation of the D01-derived T cell line by A*3101^+^ LCL and 36% of cells were stained by anti-IFN-γ mAb (not shown), thus confirming the result of the CD25 assay. In contrast, only 3% of T cells of the T cell line derived from D03 were stimulated by the LCL SBN, expressing A*3201. For the two cell lines, a fraction of CD25^+^ cells was stained by anti-Vβ13.1 mAb (TRBV6-9; Fig. 3B), while a fraction of CD25^+^ cells was not stained by this mAb, nor by any of the other anti-Vβ mAb (data not shown). Due to the down-modulation of the TCR-CD3 complex following activation by Ag, most probably the whole fraction of CD25^+^ T cells activated by the allogeneic HLA molecule is included in the TRBV6-9 subset. This suggests that alloreactive T cells contained in the pp65/A2-specific T cell lines from D01 and D03 used the same TCR Vβ (TRBV6-9), though they did not recognize the same allogeneic HLA allele.

### Allorecognition of HLA-Cw*0602 by T cells specific to the BZLF1_54–64_/B*3501 epitope

Strong allorecognition of HLA-Cw*0602 was observed for the BZLF1/B*3501-sorted T cell line from D15. This T cell line killed selectively the four LCL of the panel (BAR, MHE, SBN, and TIM), that shared Cw*0602 expression (Fig. 2A) and produced TNF-α selectively against COS cells expressing Cw*0602 (Fig. 2B). The CD25 assay, performed as described above, indicated that at least 70% of T cells within this cell line acquired CD25 expression upon stimulation with Cw*0602^+^ LCL, but not with Cw*0602^−^ LCL (Fig. 3C), thus indicating that T cells that recognize an EBV epitope in the B*3501 context can cross-react with an HLA-C allele. The two other BZLF1_54–64_/B*3501-sorted T cell lines did not respond to Cw*0602, though they were stained at >90% by the BZLF1_54–64_/B*3501 tetramer (Table 1). All three BZLF1/B35-sorted T cell lines killed B*3501^+^ LCL loaded with the BZLF1_54–64_ peptide (data not shown).

Analysis of TCR Vβ repertoire of BZLF1/B*3501-sorted T cell lines showed a limited set of TCR Vβ regions, with a preferential usage of Vβ12 (TRBV10-3) for the three cell lines (Table 3). Repertoire skewing in favor of Vβ12 expression was the most striking in D15-derived T cell line, since almost all T cells use this Vβ region gene. The two other BZLF1/B35-sorted T cell lines comprised about 50% of Vβ12 T cells, though they did not exhibit allorecognition of Cw*0602, thus indicating that alloreactive T cells within D15-derived T cell line correspond to specific clonotype(s) not found in the two other cell lines.

### Alloreactive CD8 T cells killed efficiently endothelial cells expressing the relevant class I HLA molecules

**Figure 4:**
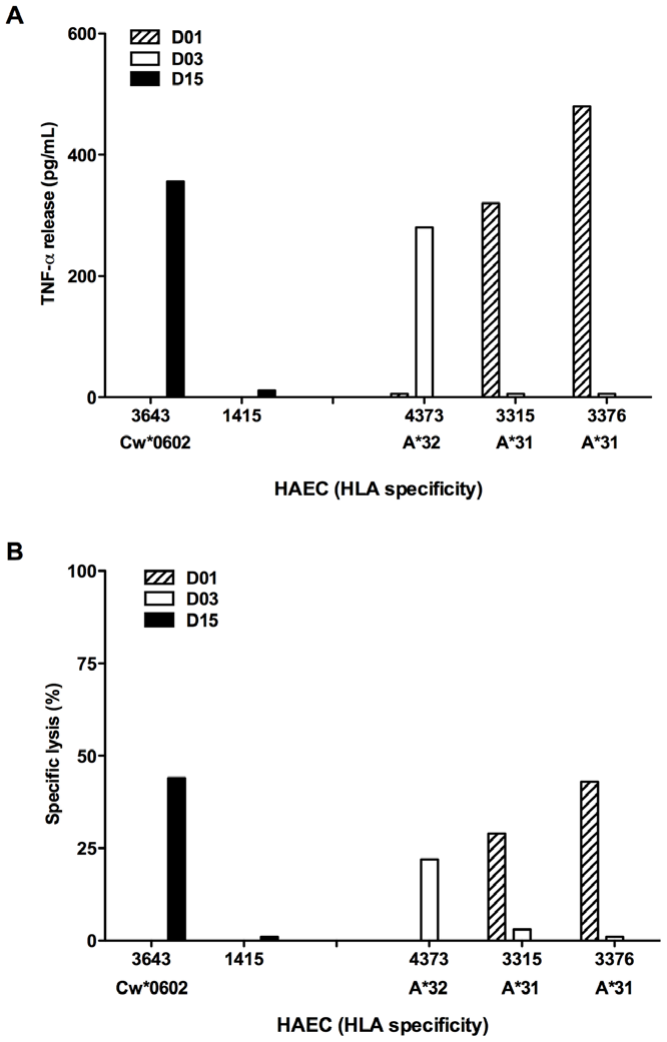
Allorecognition of human endothelial cell cultures by CD8 T cell lines enriched in herpesvirus-specific T cells. T cell lines from D01 and D03, sorted with pp65_495–503_/A*0201 multimers, were tested against HAEC #4373, #3315, and #3376, while T cell line from D15, sorted with BZLF1_54–64_/B*3501 multimers, was tested against HAEC #3643 (Cw*0602+) and #1415 (Cw*0602-) for TNF-α production (**A**) and cytotoxicity (Effector-Target ratio 15∶1) (**B**). TNF-α production was measured as indicated in Figure 2 legend. Only the endothelial cell lines expressing the relevant allogeneic HLA allele induced cytotoxicity and TNF production by the CD8 T cell lines tested. The data are representative of 3 different experiments.

Endothelial cell cultures were tested for their capacity to activate alloreactive CD8 T cell lines. As endothelial cell cultures, expressing A*31, A*32 or Cw*0602 were available, we tested their capacity to activate CD8 T cell lines that had shown alloreactivity toward these HLA alleles: T cell line from D01, alloreactive to A*3101, T cell line from D03, alloreactive to A*3201 and T cell line from D15, alloreactive to Cw*0602. While endothelial cell cultures alone did not produce TNF-α, they induced TNF-α responses from T cell lines specific for the relevant allogeneic MHC allele (Fig. 4A). Moreover, alloreactive CD8 T cell lines killed the endothelial cells expressing the relevant allogeneic MHC molecules, but not endothelial cells with irrelevant allogeneic MHC alleles (Fig. 4B and Table 4).

**Table 4:** HLA class I typing of endothelial cells.

Endothelial cells	HLA-A*		HLA-B*		HLA-Cw*	
HAEC 1415	01	02	07	08	0702	
HAEC 3315	30	31	37	39	ND	
HAEC 3376	24	31	35	60	ND	
HAEC 3643	01	02	15	57	0602	
HAEC 4373	02	32	44	55	ND	

ND : Not determined.

## Discussion

We identified in this study the cross-reactivity of herpesvirus-specific CD8 T cell lines with allogeneic MHC class I molecules. The rationale for analyzing these particular HCMV and EBV epitopes was that their immunodominance has been well documented [17], [19], [22]. CD8 T cell lines were screened on a large panel of class I HLA alleles expressed either by LCL or by COS-7 cells transfected with HLA-encoding cDNA. Identical results were obtained in both assays, thus validating the use of COS cells transfected with individual HLA allele to screen for allorecognition in the MHC class I context. The interest to use T cell lines sorted out with recombinant p/MHC multimeric complexes was that they were 90 to 100%-enriched for T cells of a given specificity, so that we could clearly link alloresponse to a given viral epitope.

In agreement with a recent report by Amir et al. [26], our data bring evidence that the pp65/A2 T cell response contributes to the alloreactive repertoire. Cross-recognition of allogeneic HLA-A molecules was found for three out of the eleven T cell lines tested that showed cytolytic activity and cytokine production toward cells expressing the relevant allogeneic HLA allele. This is in accordance with previous data, obtained with EBV-specific CD8 T cells, which showed that allogeneic responses are considerably stronger than syngeneic responses [12], [13], [14]. In contrast to the strong cytotoxicity toward allogeneic LCLs exhibited by the T cell lines we analysed, the pp65/A*0201-specific CD8 T cells cross-reactive to allogeneic molecules described by Gamadia et al. were not able to kill allogeneic LCLs, though they proliferated and produced IFN-γ against allogeneic LCL and killed autologous LCL loaded with the pp65/A2 peptide [15]. We did not find any recurrent cross-reactivity to allogeneic MHC alleles as each one of these three alloreactive T cell lines recognized a different allogeneic HLA-A allele: A*3001, A*3101, or A*3201. Remarkably, the pp65/A2-specific T cell line from D08 that showed alloreactivity toward A*3001 did not respond to A*3002, though these two allotypes differ from each other by only four amino acid within α2 and α3 domains, indicating exquisite HLA specificity for this allorecognition. Despite lack of recurrence, alloreactivity of pp65/A2-specific T cells was focused against three HLA-A alleles, that share >95% homology between each other and with HLA-A*0201. This is in accordance with a retrospective survey of a large cohort of hemopoietic stem cell donor/patient pairs with single HLA mismatch, that had shown that allogeneic HLA class I molecules with large sequence differences do not elicit a CTL response [27]. In light of the A*3001, A*3101 and A*3201-cross-recognition by pp65/A2-specific T cell lines, we tried to determine retrospectively whether single HLA-A2-A30, A2-A31, and A2-A32 mismatch combinations could be associated with increased renal graft loss [28] or higher incidence of severe GVHD [29]. However no conclusive results could be drawn yet from such an analysis, owing to the scarcity of such alleles, which are expressed by at most 4% of the caucasian population.

We could have missed alloreactive clonotypes poorly represented within the polyclonal T cell line. However, our data indicate that when as few as 3% of alloreactive T cells were present in the polyclonal population, alloresponse was detected, thus documenting the exquisite sensitivity of the functional assays herein used. In accordance with previous studies [17], [24], [25], most pp65/A*0201-specific CD8 T cell lines screened for allorecognition showed a diversified TCR Vβ repertoire, so that a large number of clonotypes were screened concomitantly. Though alloreactive T cells contained in the pp65/A2-specific T cell lines from D01 and D03 used the same TCR Vβ (Vβ13.1), they did not recognize the same allogeneic HLA allele. Moreover, despite pp65/A2-specific T cell lines from D04 and D11 used preferentially the same TCR Vβ (>90% TRBV6-9+ T cell), no alloreactivity was observed. Collectively, these results suggest that alloreactivity exhibited by some of the pp65/A2-specific T cell lines analyzed in this study was linked to particular clonotypes.

Though several HLA-C locus mismatch combinations have been described to be significant risk factors for severe acute graft-versus-host disease GVHD [29], allorecognition of HLA-Cw alleles has been poorly documented until now [29], [30]. This study shows allorecognition of HLA-Cw*0602 by a BZLF1_54–64_/B*3501-sorted T cell line, and this is, to our knowledge, the first example of cross-recognition of an HLA-Cw allele by HLA-B-restricted T cells. Cross-reactivity to Cw*0602 was observed in only one BZLF1/B*3501-specific T cell line out of three, thus indicating that alloreactivity is mediated by private T cell clonotype(s). It has been reported that a recurrent CDR3 sequence using TRBV10-3 is found in CTL recognizing BZLF1_54–64_/B*3501 [23]. Thus, the bulge of the EPLP 11-mer has been postulated to constrain TCR diversity. As a similar predominant use of TRBV10-3 has been observed in the 3 analyzed BZLF1_54–64_/B*3501-sorted T cell lines but only one exhibit a cross-reactivity to Cw*0602, it would be of interest to characterize the CDR3 sequences in order to determine if a minor difference in TCR sequence influence the allo-cross-reactivity with HLA-Cw*0602.

Again, this underlies the difficulty to predict alloreactivity, given the huge number of HLA alleles and diversity of clonotypes directed against a particular p/MHC complex. Analysis of alloreactive T cell populations infiltrating human allografts undergoing rejection have shown highly biased TCR usage, associated in some cases with predominance of a single clone [31]. Alloreactive T cell clonal expansions have also been identified *in vivo* during acute GVHD and persistence of these T cells for up to one year has been reported [32]. Although the basis for this limited diversity is unclear, it is possible that pre-existing expansions of primed alloreactive T cells could play a role in graft rejection or GVHD.

A range of acute viral infections have been linked with initiation of complications that often follow transplantation, and most attention has focused on the role of herpesvirus infections [1], [2], [3], [33]. In this context, the influence of the public CD8 T cell response to the EBNA3A_325–333_/B*0801 EBV epitope on the allogeneic repertoire has been well documented [12], [13], [14]. Clones were isolated, that displayed dual specificity for the EBNA3A_325–333_/B8 epitope and for HLA-B*14, B*44 or B*35 alleles, as alloantigens [12], [13]. Each distinct pattern was found to be associated with a public TCR. The public TCR associated with B44 allorecognition was shown to be alloreactive against B*4402 and B*4405, but not B*4403 [12], [33]. These three allotypes differ from each other by only 1 or 2 amino acids; yet this difference is enough to invoke a substantial difference in T cell recognition. These reports were the first to demonstrate that a history of EBV infection can augment responsiveness to particular alloantigens. Interestingly, HLA-B44 was identified as a ‘taboo mismatch’ for HLA-B8^+^ renal transplant recipients [34].

While it has long been established that human vascular endothelium can activate alloreactive CD8 T cell *in vitro* [35] very few studies have examined in detail the response of alloreactive CD8 T cells toward endothelial cells. Here we show that alloreactive CD8 T cell lines efficiently killed endothelial cells expressing the relevant allogeneic HLA allele, and produced high level TNF-α in response to these allogeneic endothelial cells. These data clearly indicate that endothelial cells cultivated *in vitro* are targets for alloreactive CD8^+^ T cells. Allograft rejection often involves injury of graft endothelium lining both large and small vessels. Human vascular endothelial cells display both class I and class II MHC molecules and are directly recognized *in vitro* by CD8^+^ and CD4^+^ alloreactive T cells, respectively [35], [36], [37]. Moreover, experiments using immunodeficient mouse hosts have revealed that human endothelial cells are capable of triggering graft rejection by adoptively transferred alloreactive T cells [38]. This issue is important in transplantation because, unlike hemopoietic APCs, allogeneic vascular endothelium remains in the allograft indefinitely.

## Materials and Methods

### Ethics Statement

PBL from healthy donors were obtained from the blood bank (EFS Pays de la loire), which informed the healthy donors about the final use of their blood. Based on their choice regarding the destination of their blood (research vs. medical purpose), healthy donors signed a consent statement. The approval of an ethical committee was thus not necessary. A signed convention was established between our institution (INSERM) and the blood bank (EFS Pays de la Loire) to have access to PBL from healthy donors for research purpose.

### T cell lines and culture

T cell lines were derived from PBL, originating either from healthy donors (D01 to D08, and D12) or from patients suffering from arthritis (D09 to D11, and D13 to D16) that were recruited by the department of Rheumatology (Centre Hospitalier de l'Université de Nantes, Nantes, France), as previously described [20]. PBMC were separated by Ficoll density centrifugation (LMS Eurobio). HLA class I genotyping of donors (Table 1) was performed by the Etablissement Français du Sang (Nantes, France).

T cells were maintained in RPMI 1640 medium supplemented with 10% FCS, 1mM L-glutamine and 150 U/mL recombinant IL-2 (hereafter referred to as IL-2/CM). CD8^+^ T lymphocytes were positively selected by magnetic cell sorting from PBL using anti-CD8 mAb, then expanded *in vitro* under nonspecific stimulation in IL-2/CM supplemented with leukoagglutinin at 1 mg/mL, irradiated allogeneic PBL and LCL as described [39]. T cells were maintained for at least 3 weeks without restimulation.

### Target cells

A panel of thirty LCL was used. They were generated by exogenous transformation of peripheral B cells with EBV-containing supernatant from the virus-producing B95.8 marmoset cell line and were cultured in RPMI medium supplemented with 10% fetal calf serum (FCS). Human arterial endothelial cells (HAEC) were isolated from renal artery patches collected at the time of kidney transplantation, harvested according to good medical practice and stored in the DIVAT Biocollection (French Health Minister Project n°02G55), and cultured as described previously [40]. All target cells were typed by HLA class I DNA sequencing (Table 2 and 4).

### Immunomagnetic cell sorting

The following antigenic peptides were used: pp65_495–503_/A*0201 from the HCMV pp65 protein; BZLF1_54–64_/B*3501, BMLF1_259–267_/A*0201, from lytic EBV proteins (Genosys).

Soluble pMHC monomers were synthesized as previously described [41], [42]. They comprised mutated HLA I heavy chains, with an Ala to Val substitution in the α3 domain at position 245, decreasing the affinity for the CD8 co-receptor. Sorting of antigen-specific T cells using recombinant pMHC complexes loaded onto streptavidin-coated magnetic microbeads was performed on CD8-sorted T lymphocytes, as described [41], [42]. T cells were then expanded *in vitro* under non-specific stimulation, as described above, and maintained for 3 weeks without restimulation before analysis. The purity of Ag-specific T cells after one sorting was checked by tetramer staining. No major perturbation in the TCR Vβ repertoire is induced by the culture system [43].

### Flow cytometric analysis

T cell lines were phenotyped for 24 TCR Vβ expression (IOTest Beta Mark PN IM3497) and anti-CD3-PE-Cy5 mAb (Beckman Coulter). Staining with pp65_495–503_/A*0201, BZLF1_54–64_/B*3501 or BMLF1_259–267_/A*0201 tetramers was performed at 4°C with PE-labeled pMHC complexes at 10 mg/mL and anti-CD3-FITC mAb (Beckman Coulter) for 30 minutes at 4°C, as described previously [41], [42]. Double stained cells were analyzed by flow cytometry (BD FACSCalibur; Becton Dickinson). Data analysis was performed using CellQuest Pro software (Becton Dickinson).

### Plasmids

Plasmids encoding the following HLA antigens were included in the analysis: A*0101, A*0201, A*0301, A*1101, A*2402, A*3201, A*3402, A*6801, B*0702, B*0801, B*1302, B*1401, B*1516, B*1801, B*2701, B*2702, B*2704, B*2705, B*2706, B*2709, B*3501, B*3508, B*4001, B*4002, B*4402, B*4403, Cw*0102, Cw*02021, Cw*0302, Cw*0303, Cw*0401, Cw*0501, Cw*0602, Cw*0701, Cw*0702, Cw*0802, Cw*1402, Cw*1501, Cw*1601. Plasmids encoding BMLF1, BZLF1, and pp65 were also used.

### Transient transfection of COS-7 cells and TNF-α assay

Transfection of COS-7 cells was performed by the DEAE-dextran-chloroquine method, as described [20]. COS-7 cells were either transfected with 100 ng of cDNA encoding one of the 39 HLA class I-encoding cDNAs, that were available, or cotransfected with 100 ng of an expression vector encoding EBV or CMV protein and 100 ng of an expression vector encoding the restricting HLA allele. Transfected COS-7 cells were tested 48 h after transfection for their ability to stimulate the production of TNF-α by CD8 T cell lines. Briefly, 5×10^3^ cells from a CD8 T cell line were added to transfected COS-7 cells, culture supernatants were harvested 6 h later and their TNF-α content was determined by measuring their cytotoxicity on WEHI-164 clone 13 cells in a MTT colorimetric assay [44], [45]}.

### Lymphocyte functional assays

Cytotoxicity of T cell lines against LCL or primary cultures of endothelial cells was evaluated in a standard 4h ^51^Cr-release assay, as previously described [46]. When used, synthetic peptides were directly added to ^51^Cr-labeled targets and incubated for 1 hour before excess unbound peptide was washed off. Results are expressed as percent specific ^51^Cr lysis = (experimental release - spontaneous release)/(maximum release - spontaneous release)×100%. Maximum and spontaneous release were determined by respectively adding 1% Triton X-100 or medium to target cells in the absence of effector cells. Each test was performed in triplicate at the indicated E∶T ratio.

In TNF-α release assays, 5×10^3^ T cells were incubated with cultured HAEC (3×10^4^ cells/well) generated from different donors and the amount of TNF-a released in the supernatant was estimated after 6 hours by the WEHI-164 cytoxicity assay [44].

To assess the percentage of alloreactive T cells within a given T cell line, T cells (1.5×10^5^ cells/well) were stimulated overnight with allogeneic LCL cells expressing or not the relevant allogeneic HLA allele (E∶T = 1∶1). Sixteen hours later, cells were double stained by anti-CD25-FITC (BD Pharmingen) and anti-CD3-APC mAb (Beckman Coulter), and analyzed by flow cytometry using a BD FACSCalibur (Becton Dickinson, san Jose, CA). To determine the TCR Vβ region used by the fraction of alloreactive T cells, the same protocol of stimulation with allogeneic LCL was followed and cells were triple stained by anti-CD3-APC, anti-CD25-FITC and by anti-Vβ-PE mAb specific to each one of the TCR Vβ used by those T cell lines, (Beckman Coulter).
